# Schober test is not a valid assessment tool for lumbar mobility

**DOI:** 10.1038/s41598-024-54787-2

**Published:** 2024-03-05

**Authors:** Nima Taheri, Luis Becker, Sandra Reitmaier, Maximilian Muellner, Friederike Schömig, Matthias Pumberger, Hendrik Schmidt

**Affiliations:** 1 Center for Musculoskeletal Surgery, Charité-Universitätsmedizin Berlin, Corporate Member of Freie Universität Berlin, Humboldt-Universität zu Berlin, Charitéplatz 1, 10117 Berlin, Germany; 2 Berlin Institute of Health, Julius Wolff Institute for Biomechanics and Musculoskeletal Regeneration, Charité-Universitätsmedizin Berlin, Berlin, Germany

**Keywords:** Rheumatology, Musculoskeletal system, Anatomy, Musculoskeletal system

## Abstract

The Schober test is considered reliable in evaluating lumbar mobility and its impairment. Especially in patients with chronic low back pain (cLBP) identification of functional restriction is important. We aimed to investigate whether the 5 cm Schober cut-off provides a valid distinction between unrestricted and restricted mobility in participants with and without cLBP (18–65 years). cLBP is defined as LBP persisting for ≥ 12 weeks. We analyzed agreement between the Schober test with two measurement devices (Epionics SPINE^®^; Idiag M360^®^) and the influence of lumbar lordosis (LL) on their agreement. Also, the sensitivity and specificity of the Schober test was evaluated. For 187 participants (49.6%) Epionics SPINE^®^ RoF and Schober test matched (either ≥ 5 cm; > 40.8° RoF or ≤ 5 cm; < 40.8° RoF), for 190 participants (50.4%) the two measurements did not. Idiag M360^®^ RoF of 190 participants (50.4%) showed corresponding results (either ≥ 5 cm; > 46.0° RoF or ≤ 5 cm; < 46.0° RoF). Non-agreement was seen in 187 participants (49.6%). LL differed significantly in the Epionics SPINE^®^ cohort (p < 0.001). Regarding the Epionics SPINE^®^ cohort, Schober test showed a sensitivity of 79.6% with a specificity of 36.1%. For the Idiag M360^®^ cohort, Schober test showed a sensitivity of 68.2% and a specificity of 46.6%. Our results do not establish a consistent matching between Schober test and the device measurements. Therefore, Schober test may not be valid to predict impairment of lumbar mobility. We recommend Schober test as an add-on in monitoring of an individual relative to its case.

## Introduction

Low back pain (LBP) has a lifetime prevalence of up to 80%^1^ of which 4–25% become chronic depending on age and sex^2^. Globally, it is the leading cause of years lived with disability^3^ and leads to a high quota of work absence, loss of productivity, and high rates of hospital admissions that result in tremendous direct and indirect costs for societies’ healthcare systems and economies.

The assessment of the spinal function is a basic component of the physical examination of LBP patients^4,5^, where the range of motion (RoM) of the spine is a common outcome parameter^6^. This notion is due to the assumption that correcting motion aberrations and restoring functional capacity can reduce pain^4,7^. Reliable and reproducible mobility tests are essential to assess back function^8,9^. Current clinical examinations typically include basic kinematic assessments, including the RoM in flexion, extension, lateral bending, and/or axial rotation as well as tests for lumbar flexibility. Examinations of lumbar flexibility may also include more specific tests as the occiput-to-wall distance, tip-toe stand, heel stand or the finger-to-floor distance^10^. For lumbar mobility the Schober test^11,12^ is considered to be an efficient diagnostic tool. Historically, the Schober test has proven to be a reliable measure in the quantification of lumbar flexibility and is used in the diagnosis of LBP as well as in rheumatological diagnostics to quantify loss of motion in axial spondylarthritis^13–17^. While the patient is in a standing position, the examiner marks the spinous process of the first sacral vertebra and 10 cm above this point. Then the patient is instructed to flex forward as if attempting to touch his/her toes while keeping the knees straight. If the distance of the two points does not increase by at least 5 cm, this is evaluated as a sign of restriction in the lumbar flexion^16^. Due to concerns that the conventional Schober test may fail to evaluate movement of the whole lumbar spine^11^, the test was changed leading to two modified versions: the modified Schober test^18^ and the modified-modified Schober test^19^. Over the years, contradicting evidence regarding the validity and reliability of each of these tests has been published when comparing it to inclinometer measurements^20–22^ or radiological analysis^23,24^.

The first description of the Schober test dates back to the 1930s^12^. Demographically and anthropometrically, society has evolved significantly since then; therefore, it is questionable whether the 5 cm cut-off of the Schober test is a valid predictor for identifying patients with unrestricted or restricted lumbar mobility. Also, the Schober test neglects the initial shape of the back in the upright standing position, e.g., flat back vs. strongly lordotic back. Our research group assumes different lumbar shapes throughout different subgroups of the population, for which a “one fits all” cut-off does not truly indicate the extent of mobility. Therefore, the present study aimed to investigate the extent to which the 5 cm cut-off of the Schober test provides a valid distinction between unrestricted and restricted mobility in a population with and without chronic LBP (cLBP). For this purpose, we performed the Schober test and a measurement with a non-invasive device on two study groups simultaneously; the Schober test and the Epionics SPINE^®^ in one study group and the Schober test and Idiag M360^®^ measurement in the other group. In contrast to the Schober test, the two non-invasive devices consider the initial shape of the back and record the shape and movement of the back. Non-invasive measurement devices have the potential to facilitate the assessment of spinal shape and RoM, nevertheless, they are not routinely used in clinical and research settings. We hypothesized that the measurement of the Schober test does not correspond with the mobility measurements of the Epionics SPINE^®^ or Idiag M360^®^ device and therefore the 5 cm cut-off of the Schober test is invalid to distinguish between unrestricted and restricted mobility.

## Materials and methods

### Study participants

#### Propensity-score matching

Significant differences were detected in between the two cohorts, therefore matching according to age, sex, body height and BMI was performed, which are known to be influencing factors for lumbar mobility as well as on Schober test. Propensity-score matching between cLBP-patients and control group was performed for the above mentioned parameters with a matching factor of 0.01. After matching, both study populations consisted of 377 patients. The demographic and anthropometric details are given in Tables 1 and 2.

**Table 1:** Demographics and anthropometrics of the included participants in the **(A)** Epionics SPINE^®^ and (**B)** Idiag M360^®^ cohort after matching.

	LBP patients (n = 111)		Asymptomatic individuals (n = 266)	
	Male Mean (SD)	Female Mean (SD)	Male Mean (SD)	Female Mean (SD)
(A)				
N	44	67	124	142
Age (years)	46.6 (12.7)	49.1 (12.9)	39.8 (13.3)	41.4 (14.4)
Height (cm)	178.1(6.6)	167.8 (6.0)	180.1 (8.0)	166.7 (6.8)
BMI (kg/m^2^)	24.7 (1.9)	23.6 (3.0)	24.4 (1.6)	23.3 (1.9)
(B)				
N	44	67	124	142
Age (years)	45.0 (11.2)	47.0 (11.3)	40.6 (12.2)	41.5 (13.1)
Height (cm)	180.2(6.0)	167.5 (6.7)	180.2 (6.0)	167.9 (6.7)
BMI (kg/m^2^)	7.1 (2.2)	22.4 (2.4)	23.8 (7.1)	22.4 (2.2)

*SD* standard deviation.

**Table 2:** Lumbar lordosis measured by Epionics SPINE^®^ and Idiag M360^®    ^

	Schober = RoF Lumbar lordosis (°)	Schober ≠ RoF Lumbar lordosis (°)	p value
(a) Epionics SPINE^®^	28.24° ± 10.97°	31.57° ± 8.51°	< 0.001
18–30 years	36.19° ± 9.31°	34.28° ± 7.68°	0.14
31–40 years	28.85° ± 11.5°	31.95° ± 9.65°	0.11
41–50 years	26.68° ± 11.21°	29.29° ± 7.48°	0.09
51–65 years	23.33° ± 9.19°	28.21° ± 9.13°	0.005
(b) Idiag M360^®^	28.74° ± 9.51°	28.32° ± 8.22°	0.329
18–30 years	31.60° ± 3.59°	30.18° ± 8.49°	0.25
31–40 years	27.26° ± 9.00°	27.10° ± 7.2°	0.46
41–50 years	30.95° ± 26.7°	26.70° ± 8.85°	0.01
51–65 years	26.09 ± 28.97°	28.97° ± 8.13°	0.04

### Evaluation of lumbar flexibility

#### Schober test

The clinical examination was performed by two orthopedic residents with multiple years of clinical experience. The spinous process of the first sacral vertebra was palpated and marked by a skin pen, then in an upright standing position, a point 10 cm cranial was located by measuring tape and marked. Subsequently, all study participants were asked to perform a full trunk flexion with extended knees shoulder with apart. After reaching the final position, the distance between the two marks was measured with the help of a tape measure and the amount of displacement was noted as the difference of the measurement value subtracted by10 cm.

#### Epionics SPINE^®^ measurements

**Figure 1:**
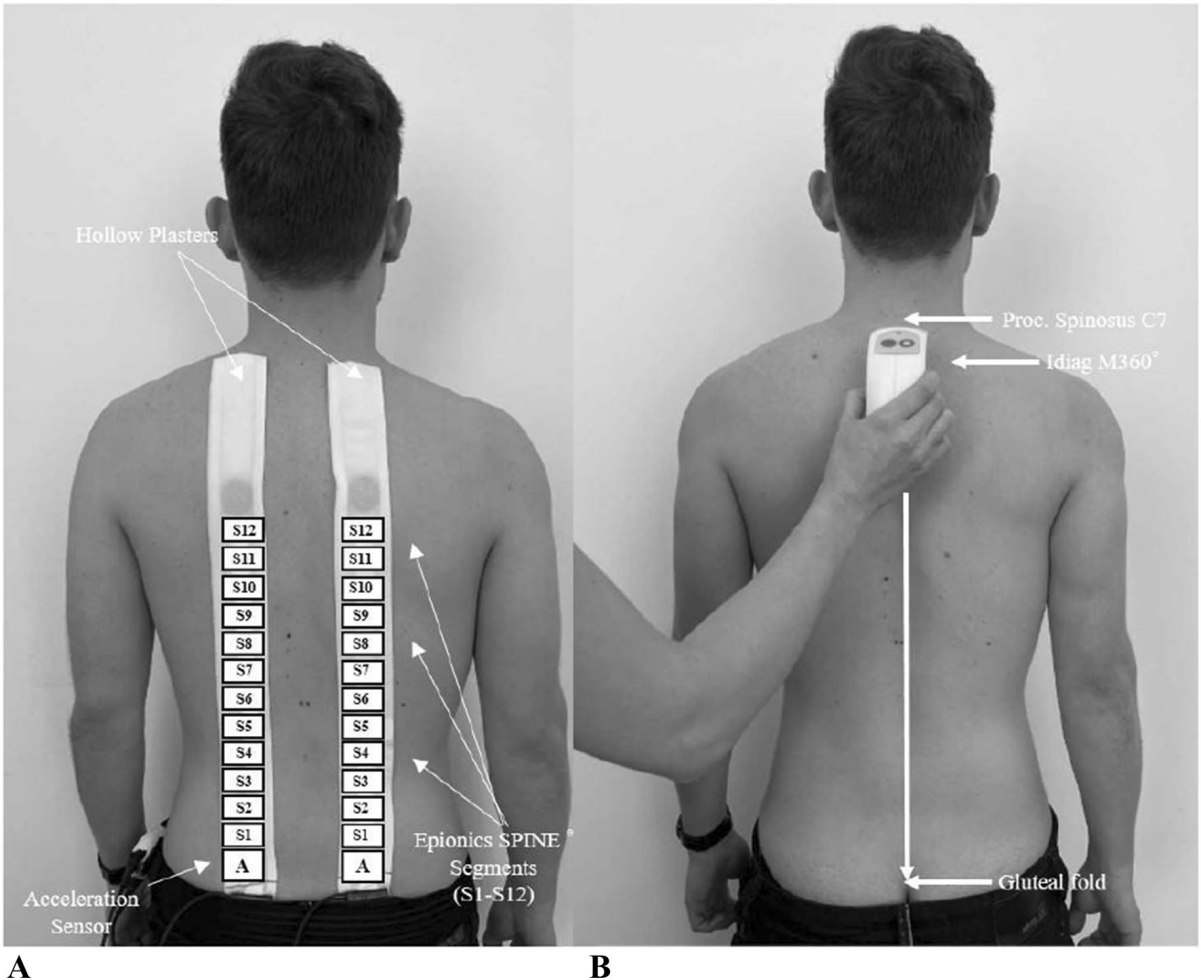
Visual representation of the The Epionics SPINE^®^ system and Idiag M360^®^ measurement. (**A**) Epionics SPINE^®^ affixed to a volunteer’s back in standing. The system consists of two flexible sensor strips utilizing strain-gauge sensors, tri-axial accelerometers and a storage unit. The total lordosis angle is the sum of all lordotically curved Epionics SPINE^®^ segments during standing. (**B**) Measurement with Idiag M360^®^ from the spinous process of the seventh cervical vertebrae until the gluteal fold.

The Epionics SPINE^®^ system (Epionics Medical GmbH, Potsdam, Germany) was used to assess lumbar spinal shape in an upright standing position as well as lumbar flexibility in the sagittal plane (Fig. 1A). The system consists of two flexible sensor strips, each of them contain twelve 2.5-cm-long segments based on differential strain-gauge elements, which provide a sensitive measure of the curvature in each segment. During a measurement, the sensor strips are inserted into two hollow plasters attached to the back paravertebrally, 7.5 cm away from the spinal column on each side. The lower end of each strip was aligned with the posterior superior iliac spine, which was approximately in line with the first sacral vertebra. A tri-axial accelerometer was located at this end, allowing the system to assess sacral orientation. This acceleration sensor determined the spatial orientation of the sensor relative to the vertical direction of the earth’s gravitational field. The sensor strips were connected to a storage unit (size: 12.5 cm × 5.5 cm; mass: 80 g) that collected data with a frequency of 50 Hz. The sensor strips of the system exhibit high accuracy and repeatability (interclass correlation coefficient, ICC > 0.98) with test–retest reliability ICCs of > 0.98. Previous studies confirmed the suitability of the system for assessing lumbar and pelvic motion^7,25^.

#### Idiag M360^®^ measurements

The Idiag M360^®^ (MediMouse, Idiag AG, Fehraltorf, Switzerland) is a hand-held device that allows evaluation of spinal shape. The shape of the spine, which is measured on two rolling wheels, is transmitted to a computer via a Bluetooth connection. The device is rolled from the spinous process of the seventh cervical vertebra through the spinous process to the anal fold (Fig. 1B). For reproducible measurements, the spinous process of the seventh cervical vertebra and at a reference point 2 cm below the junction of the left and right posterior superior iliac spines are marked. During the measurement, lordosis angles of the individual vertebral bodies are determined. After performing the Schober test, back shape was measured with the Idiag M360^®^ in an upright position and in full flexion. The validity and reliability were established in previous studies^26–29^.

### Measurement protocol

In the first step, the Schober test, the Epionics SPINE^®^ and Idiag M360^®^ measurements were performed in maximum upper body Range of Flexion (RoF) once for all study participants.

#### 1st test: measurements under full upper body flexion

All study participants of the first study group were equipped with the Epionics SPINE^®^ system. Before starting the exercise, study participants were explicitly requested to stand in a relaxed upright position with extended knees on a platform of 30 cm with marked foot position shoulder width apart. Subsequently, they performed a full flexion motion twice as a trial to get used to the examination, the third trial was evaluated. After reaching the final position, the Schober measure was taken. During the whole exercise, the back movement was recorded with the Epionics SPINE^®^ system. In the Idiag M360^®^ cohort, after marking of the reference points study participants were explicitly requested to stand in a relaxed upright position with extended knees on a platform of 30 cm with marked foot position shoulder width apart. The stance was recorded with the device. Then they performed a full flexion motion, after reaching the final position the Schober measure was taken. Afterwards, spinal shape was assessed with Idiag M360^®^ in full standing position as well as in maximum flexion.

### Correlation of Lumbar RoF to Schober test

Epionics SPINE^®^ Lumbar RoF and Idiag M360^®^ Lumbar RoF was determined as the difference of lumbar lordosis in full trunk flexion and in upright standing position measured by Epionics SPINE^®^ system and Idiag M360^®^, respectively. Agreement with Schober values was assessed as follows: a low Schober value accompanied by low Epionics SPINE^®^ or Idiag M360^®^ Lumbar RoF and vice versa a high Schober value accompanied by a high Epionics SPINE^®^ or Idiag M360^®^ Lumbar RoF, respectively, represented agreement. Cut-off values for low Epionics Lumbar RoF were determined by lowest 16% of the values of the asymptomatic participants, therefore exceeding more than a standard deviation (SD) from the mean. The evaluation was performed for both study groups.

To analyze the influence of age on the agreement between the respective non-invasive device measurement and Schober test, study participants were assigned into the following age groups for a separate analysis: 18–30 years, 31–40 years, 41–50 years, and 51–65 years.

Afterwards, we considered whether lumbar lordosis in upright stance is the factor yielding agreement or non-agreement between Schober test and device measurements. The influence of lumbar lordosis was also analyzed depending on age.

### Statistical analysis

Data analysis was performed using SPSS Version 27 (IBM Corp. Released 2020. IBM SPSS Statistics for Windows, Version 27.0. Armonk, NY: IBM Corp). To evaluate a normal distribution of the values, a Kolmogorov–Smirnov test was performed. For the comparison of unpaired parametric samples, the t-test was used, and for non-parametric samples, the Mann–Whitney-U test was used. For the comparison of two paired parametric samples, the paired t-test was used, and for non-parametric samples, the Wilcoxon rank-sum test was used. Furthermore, we analyzed sensitivity for true positive rate, specificity for the true negative rate and the false-positive rate to assess the probability of falsely specifying lumbar mobility as restricted when in truth it is unrestricted. This analysis was performed for the above mentioned age groups. Continuous data is presented in mean and its standard deviation (SD). A p value < 0.05 was considered statistically significant.

### Ethics approval and study cohorts

Both studies were approved by the local ethics committee (Epionics SPINE^®^: EA4/011/10; Idiag M360^®^: EA1/058/21) and conducted in strict adherence to the STROBE guidelines for observational studies. All study participants were informed about the study’s procedure and signed a consent form.

We included participants with a body mass index (BMI) lower than 29 kg/m^2^. Several studies have demonstrated that the shape and motion measured on the back skin surface and the spine itself significantly correlate with each other^30–32^. However, our own validation studies found that this correlation is poor in overweight and obese persons, thus the strict adherence to BMI. Further inclusion criteria were an age of at least 18 and max. 65 years with or without a history of cLBP persisting for more than 12 weeks. Patients were excluded if they had acute LBP lasting less than 12 weeks, neurological movement disorders, radiculopathy, and systemic diseases with medication such as immunosuppressants, or malignant diseases. By adherence to the in- and exclusion criteria, a total of 523 participants were eligible for this study in the Epionics SPINE^®^ cohort. Of these, 167 represented the chronic LBP patients and 356 the asymptomatic population. Regarding the Idiag M360^®^ cohort, a total of 559 patients were eligible for this study. Demographic and anthropometric details are given in Table 1A.

### Informed consent for publishing of patient images

As we have included an image that can lead to patient identification due to open-access publication, we hereby assure, that informed consent was obtained from the individual prior to publication.

## Results

### Agreement between Epionics SPINE^®^ and Idiag M360^®^ Lumbar RoF and Schober test:

#### Agreement in matched Epionics SPINE^®^ and Idiag M360^®^ cohorts

**Figure 2:**
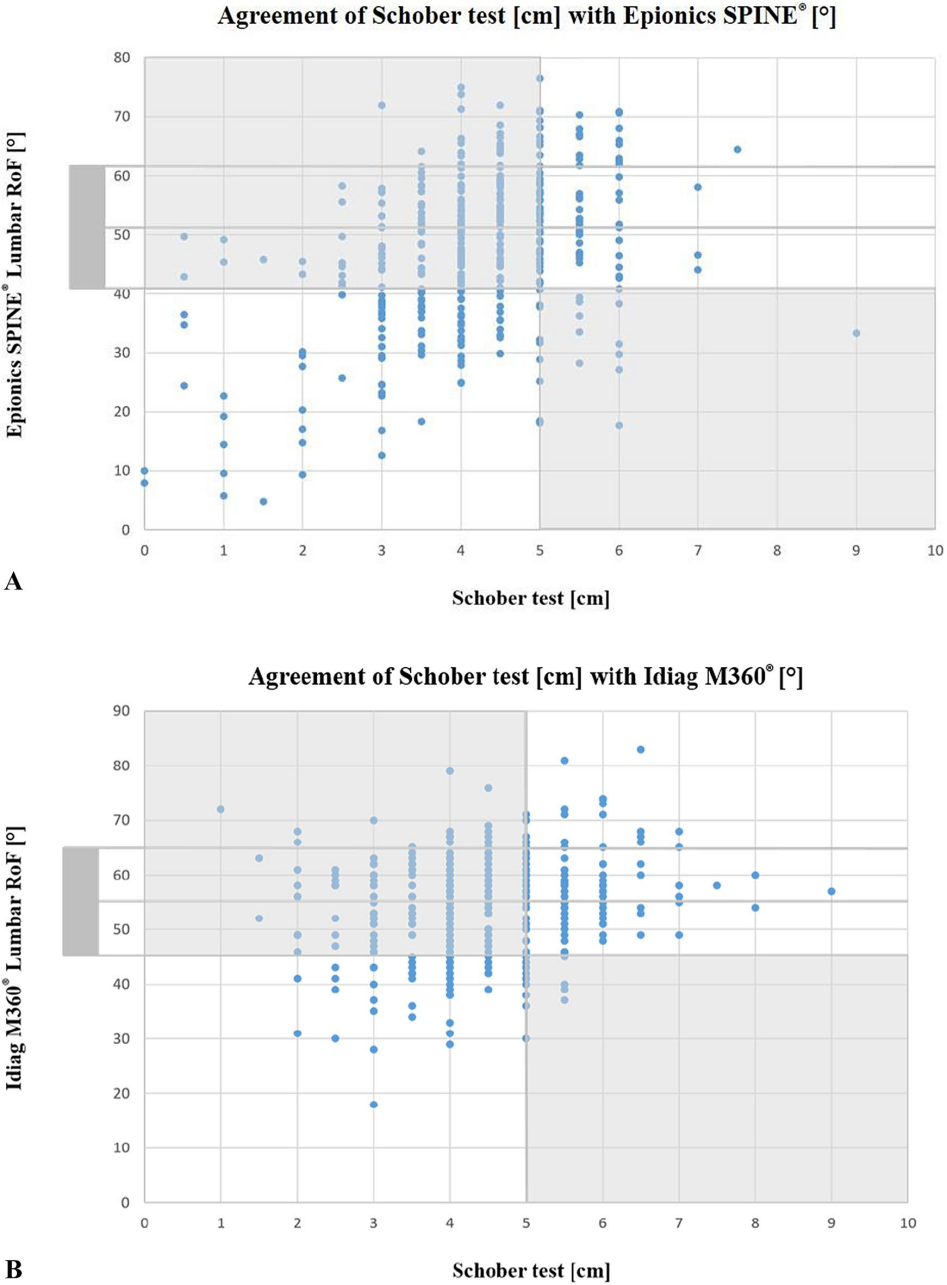
Agreement of the non-invasive devicement measurement with Schober test. (**A**) Agreement of Schober (cm) test with Epionics SPINE^®^ and (**B**) Schober test (cm) with Idiag M360^®^ Lumbar RoF (°) in the matched cohorts. White area: (**A**) Epionics SPINE^®^ or (**B**) Idiag M360^®^ measurements and the Schober test indicate low mobility (lower left) or high mobility (upper right). Light gray area: results of both measurements, Epionics SPINE^®^ and Idiag M360^®^ and Schober test, do not match. *RoF* range of flexion, *cm* centimeter. RoF reference for good lumbar mobility: Epionics SPINE^®^ = 40.8°; Idiag M360^®^ = 46.04°.

For a total of 187 study participants (49.6%), the results between Epionics SPINE^®^ and Schober test matched (Fig. 2), i.e., both measurement methods indicated either a movement deficit or good mobility. A total of 87 individuals showed low mobility (< 40.8°), in Epionics SPINE^®^, while Schober test also showed values below 5 cm. 100 individuals presented high mobility in both tests. For 14 study participants low mobility was measured with Epionics SPINE^®^, while the Schober test showed a value of 5 cm. For 190 study participants (50.4%), the results between both tests did not correspond (light gray areas in Fig. 2A). While for 176 individuals a good mobility was measured with Epionics SPINE^®^, the Schober test showed values below 5 cm.

When assessing agreement between two modalities depending on age, we analyzed agreement in 41.7% and non-agreement in 57.3% for individuals between 18 and 30 years, 55.4% agreement and 44.6% non-agreement in individuals between 31 and 40 years, 56.7% agreement and 43.3% non-agreement in individuals between 41 and 50 years and lastly 38.8% agreement and 61.2% non-agreement in individuals between 51 and 65 years.

Similar results were obtained with the Idiag M360^®^. For a total of 190 study participants (50.3%), the results between Idiag M360^®^ and Schober test matched (Fig. 2B). For 187 study participants (49.7%), the results between both tests did not correspond. While for 166 individuals a good mobility (> 46.0°) was measured with Idiag M360^®^, the Schober test showed values below 5 cm. For 21 study participants, low mobility was measured with Idiag M360^®^, while the Schober test showed values exactly or above 5 cm.

Age-depending analysis of agreement between two modalities showed agreement for 52.8% and non-agreement in 46.3% of the 18–30 years old individuals, agreement in 51.7% and non-agreement in 48.3% of the 31–40 years old individuals, agreement in 54.3% and 45.7% of the cases of individuals between 41 and 50 years and lastly, agreement in 45.0% and non-agreement in 55.0% of individuals between 51 and 65 years.

### Influence of lumbar lordosis on the agreement or non-agreement of RoF and Schober test

For the Epionics SPINE^®^ measurements, there were significant differences in between lumbar lordosis yielding agreement or non-agreement between both assessment tools (Table 2). Furthermore, when grouping the individuals of the Epionics SPINE^®^ cohort by age significantly reduced lumbar lordosis seemed to yield agreement for individuals between age 41–50 years (Table 2) as well as 51–65 years.

For the Idiag M360^®^ measurement, there were no significant differences regarding lumbar lordosis in any of the subgroups. Age-depending analysis revealed significant higher lumbar lordosis for individuals between age 41–50 and significantly reduced lumbar lordosis for individuals between 51 and 65 years to be a factor yielding agreement and non-agreement (Table 2).

### Sensitivity and Specificity of Schober test for the Epionics SPINE^®^ cohort

A sensitivity of 36.1% for Schober test for reduced lumbar mobility was observed, however a weak specificity of only 79.6% was observed with a false positive rate of 63.9%. Individuals between 18 and 30 years showed sensitivity of 66.7%, specificity of 34.6% and false-positive rate of 65.4%. For age 31–40, Schober test showed sensitivity of 77.8%, specificity of 42.6% and false-positive rate of 57.4%. For age 41–50, the respective measures changed to showed sensitivity of 81.8%, specificity of 46.5% and false-positive rate of 53.5% and lastly for age 51–65 years to a sensitivity of 75%, specificity of 20% and false-positive rate of 80%.

### Sensitivity and Specificity of Schober test for the Idiag M360^®^ cohort

In the overall cohort a sensitivity of 68.2% for Schober test was observed, however a weak specificity of 46.6% was observed with a false positive rate of 53.4%. Age-depending analysis for Idiag M360^®^ revealed the following data: For individuals between 18 and 30 years, Schober test showed a sensitivity of 45.5%, specificity of 53.6% and false-positive rate of 46.4%. For individuals between 31 and 40 years, these values changed to 50%, 81.8% and 50%. For individuals between 41 and 50 years, a sensitivity of 64.3%, a specificity of 52.5% and a false-positive rate of 47.8% was observed. Lastly individuals between the ages of 51–65 years showed a sensitivity of 80%, specificity of 20% and false-positive rate of 20%.

## Discussion

The Schober test is a widely used clinical tool for assessment of lumbar mobility both in LBP patients as well as in patients with axial spondylarthritis or other rheumatologic diseases^13–17^. The aim of our study was to assess, whether the Schober test and its cut-off at 5 cm correlate with Lumbar RoF measured by non-invasive devices such as Epionics SPINE^®^ and Idiag M360^®^. We hypothesized that the 5 cm cut-off was invalid, because the Schober test neglects to take individual lumbar spine shapes into account. In agreement with our hypothesis, we were able to show that the 5 cm cut-off value did not correlate well with Lumbar RoF measured with non-invasive devices. Regardless of the invalidity of the cut-off value, we could not demonstrate the expression of the spinal shape as a determining factor for agreement of both test methods.

50.4% and 49.6% respectively of the participants in our study exhibited a mismatch between mobility in the Epionics SPINE^®^ System and Idiag M360^®^ with the Schober test. Thereby 57.3% and 44.0% of the patients showed a good mobility in the Epionics SPINE^®^ system and Idiag M360^®^, but a poor Schober test. The reliability and validity of the Epionics SPINE^®^ System in the evaluation of spinal flexion is adequately supported by evidence^7,26^. Also, the Idiag M360^®^ has been proven to be a reliable and valid tool in assessment of lumbar flexibility^28,29,33^. One study even showed its reliability in correlation to lumbar radiographs in fully upright stance and full flexion^31^. A possible disadvantage of the Epionics SPINE^®^ system in the case of the Schober test is the application of two patch strips that possibly reduce skin stretching and thus disadvantage the Schober test. With this in mind, it was important to consider a second, reliable and validated test method to provide a meaningful assessment of the utility of the Schober test. It therefore can be concluded that in the majority of cases, a high degree of lumbar mobility would be misinterpreted as restricted mobility in the Schober test. Consequently, our results show a relatively high sensitivity for the detection of movement deficiencies, however a poor specificity was detected with a high rate of false positive results for a restricted mobility in Schober test further proving our hypothesis that the cut-off of the Schober test at 5 cm is invalid for assessment of lumbar flexibility.

Our hypothesis that participants with reduced lumbar lordosis would have better agreement between Schober test and back mobility was rejected. Our results show that, in the majority of cases, lumbar lordosis is not significantly smaller in the group of individuals showing a match between both assessment tools. After matching for demographic and anthropometric characteristics and assessing the influence of spinal shape, it can be concluded that there is no obvious factor yielding agreement or non-agreement in between Schober test and lumbar mobility.

Even though widely accepted, there is controversial evidence regarding the reliability and validity of the conventional Schober test. Some argue the risk of failing at palpating and marking the anatomical landmarks exceeds the diagnostic accuracy^11^. Therefore, the modified Schober test was created^18^. With changes in the measurement procedure the goal was a more accurate display of the lumbar spine, lumbosacral junction and the lumbar mobility. Even though clinically the modified Schober test has shown good correlation with spinal movement in asymptomatic patients^11^ and has partly displayed excellent inter-tester reliability^16^. Evidence shows growing concern that modified Schober test does not represent the whole lumbar spine^34^. Stoljwik et al. has raised concern that the modified Schober test leads to overestimation of lumbar mobility through modified Schober test in patients with and without axial spondylarthritis^17^. Hershkovic et al. recently showed that the conventional Schober test fails to include at least on lumbar motion segment in a study of 25 patients. They report that the modified-modified Schober test showed a higher accuracy of assessing all lumbar segments when compared to CT scans^24^. Yet, Rezvani et al. was able to show a weak correlation of the modified Schober test and also the modified-modified Schober test with L1-S1 mobility as well as with L3-S1 mobility compared to functional X-rays^23^. In addition, it has been shown that insufficient correlation to radiographic gold standard could be due to systematic differences at end ranges of spinal flexion when using tape measures as the overlying skin begins to slide across the underlying tissue rather than continuously stretch above it^25^. Rezvani et al. also observed this phenomenon: as angular changes increased in radiographs, the distance between 2 reference points marked on the skin decreased^23^. This could further be a reason for disagreement between the Schober test and non-invasive measurement. Skin may not be stretchable enough to match lumbar flexion. This is represented well within the age-depending analysis of the agreement and non-agreement of the respective device measurement and Schober test as the agreement rate decreases in the higher age groups for both respective cohorts. We decided for the conventional Schober test in our study as it was shown to correlate well independent of the age group and potentially underlying rheumatic diseases^11,23,35,36^. Furthermore, conventional Schober test proved to be more feasible as it did not need for presentation of the gluteal cleft. The modified-modified Schober test often leads to exposition of it.

Lastly, it has also be mentioned that poor agreement between the modalities could be due to the modalities measuring different metric: while both Epionics SPINE^®^ and Idiag M360^®^ track angular changes, conventional Schober test measures lumbar mobility in metric units^21^.

This study has limitations that need to be mentioned. We solely evaluated lumbar flexion with the conventional Schober test and excluded the modifications of this test. Therefore, we cannot conclude a general assessment on the usefulness of the modifications for clinical evaluation. As this project is part of a large-scale cross-sectional study that aims to assess various dimensions of cLBP, it was not ethically justifiable to include all modifications during the clinical assessment of the participants and patients as this would be an unnatural repetition of medical procedures. We strongly believe that the Schober test in all its modifications does not provide valid information on back or spinal mobility as the one fits all method of tape measurements is insufficient at considering individual anthropometric measures.

The validity of the 5-cm cut-off of the Schober test was proven by comparing the Schober test with two devices that measure the back shape and not directly the curvature of the lumbar spine. We found a significant correlation between lumbar lordosis assessed via the back shape and radiologically assessed spinal shape only for subjects with a body mass index (BMI) < 29.0 kg/m^2^, which is in agreement with previous studies^7^. In our validation study, we could additionally see that the correlation between the back and spinal shape becomes poor in overweight and obese persons, which limits the current study to normal-weight subjects (BMI < 29.0 kg/m^2^). Therefore, to ensure a strong correlation between back shape and underlying spinal structures, only subjects with a BMI < 29.0 kg/m^2^ were included. To reevaluate the significance of our findings, future studies should also include participants with a BMI > 29 kg/m^2^.

## Conclusion

Based on our results the conventional and historically established Schober test with end-range motion comparison with given 5 cm threshold should not be used for the clinical evaluation of lumbar flexibility because its measurements have a high-rate of false positives and can therefore lead to misinterpretation. The Schober test value can mislead examiners to underestimate the actual degree of lumbar mobility. We recommend using measurement devices that continuously measure the curvature of the back at a higher resolution to obtain a more valid conclusion regarding back function.

## Data Availability

The datasets generated and analyzed during the current study are available from the corresponding author on reasonable request.

## Abbreviations

BMI: Body mass index
LBP: Low back pain
cLBP: Chronic low back pain
RoF: Range of flexion
ROM: Range of motion
